# TSSCM: A synergism-based three-step cascade model for influence maximization on large-scale social networks

**DOI:** 10.1371/journal.pone.0221271

**Published:** 2019-09-03

**Authors:** Xiaohui Zhao, Fang’ai Liu, Shuning Xing, Qianqian Wang

**Affiliations:** 1 School of Information Science & Engineering, Shandong Normal University, Jinan, China; 2 School of Mathematical Science, Shandong Normal University, Jinan, China; Nanyang Technological University, SINGAPORE

## Abstract

Identification of the most influential spreaders that maximize information propagation in social networks is a classic optimization problem, called the influence maximization (IM) problem. A reasonable diffusion model that can accurately simulate information propagation in social networks is the key step to efficiently solving the IM problem. Synergism of neighbor nodes plays an important role in information propagation dynamics. Some known diffusion models have considered the reinforcement mechanism in defining the activation threshold. Most of these models focus on the synergetic effects of nodes on their common neighbors, but the accumulation of synergism has been neglected in previous studies. Inspired by these facts, we first discuss the catalytic role of synergism in the spreading dynamics of social networks and then propose a novel diffusion model called the synergism-based three-step cascade model (TSSCM) based on the above analysis and the three-degree influence theory. Finally, we devise an algorithm for solving the IM problem based on the TSSCM. Experiments on five real large-scale social networks demonstrate the efficacy of our method, which achieves competitive results in terms of influence spreading compared to the four other algorithms tested.

## Introduction

The problem of finding the optimal set of influencers, whereby viruses, information, and epidemics propagate through network edges via interactions between individual constituents, has broad applications in a variety of network dynamics areas [1–8]. Viral marketing can inexpensively achieve large-sale product adoption through advertising with a small group of influential customers [1–3]. The financial crisis that resulted from the cascading bankruptcy of major financial institutions in 2008 caused estimated US economic losses as high as $22 trillion [9]. The immunization of structurally important persons can efficiently halt global epidemic outbreaks. The above applications have important characteristics in common, such as budget restrictions and intervention time constraints, and require efficient real-time applications of large-scale data. These features can be simplified to an optimization problem, called the influence maximization (IM) problem. IM, first studied by Domingos and Richardson et al. [10,11], is a fundamental research problem in social networks. The issue is described as follows: an online social network can be modeled as a graph with vertices representing users and edges representing the links between users. The cascade process on the network is conducted under a specified diffusion model. The IM problem is defined as finding k seed nodes in the network as the source of information propagation such that under the specified diffusion model, the scale of the cascade is maximized. The IM problem is NP-hard. Kempe, Kleinberg and Tardos [12] proposed a greedy algorithm based on Monte Carlo simulation for solving the optimization problem. The performance of the greedy algorithm reached 63% of the optimal solution, but it is not applicable to large-scale networks because it is time consuming. A series of improved algorithms, including CELF [13], NewGreedy [14] and Mixgreedy [15], were proposed to overcome the inefficiency of the greedy algorithm. Unfortunately, although these algorithms are hundreds of times more efficient than the greedy algorithm, their computational complexity remains too high to be applied to growing networks because Monte Carlo simulations are performed to approximate the influence spread of a given seed set.

Many heuristic algorithms have recently emerged. These algorithms can be grouped into two categories: algorithms based on network topology and algorithms based on propagation path. The first group of algorithms mainly use centrality measures, including high degree [15–17], random selection [18], betweenness centrality [17,19], random walk [20], k-shell [21] and community detection [22]. Chen et al. [23] proposed a degree discount algorithm based on degree centrality. In this algorithm, when a node is selected as a seed node, the degree of its neighbor nodes is discounted. Cao et al. [24] designed a core-covering algorithm based on k-shell and the influence radius. Zhu et al. [25] solved the IM problem by developing a structural hole-based algorithm, called SHIM. These algorithms, which are based exclusively on topology, decrease the running time by several orders of magnitude, but they are unstable under different networks and diffusion models. The second group of algorithms are path based, and influence spreading can be efficiently approximated without Monte Carlo simulation. In some applications, we must identify super blockers. Giant components are fragmented by removing key nodes, and propagation is blocked. The mining of the optimal immunization set is based on a diffusion model, such as the susceptible-infected-recovered model [26], the susceptible-infected-susceptible model [26], the independent cascade model [10], the linear threshold model [10] and other cascade models [4,27–30]. Wattes [4] proposed a simple model of global cascades on random networks to explain global cascades that are triggered by small initial shocks. Wei Wang et al. [27] employed the nonredundant information memory characteristic in their social contagion model, which better captured the dynamics of social contagions in the real world, and discussed the cascading process in multiple networks [28,29]. Flaviano and Hernan A [31] mapped the information spread on social networks onto an optimal percolation and presented an algorithm, called collective influence (CI), based on the weak connection between nodes to identify the minimal set of influencers. Based on this information, the authors leverage the behavior of users in real networks, including Twitter, Facebook, APS and LiveJournal, and use the CI algorithm to locate influential spreaders. The experimental results show that the optimal seed set is much smaller than those obtained by other measures. Sen et al. [32] explored CI in the linear threshold model and proposed a method based on the subcritical path to locate influential spreaders. Andrey Y. and David [33] found that the optimal deployment of the seed set resulted from the interaction between network topology and propagation dynamics. They introduced an effective framework for optimizing the maximization or minimization of propagation. Qin et al. [34] devised a diffusion model, called the three-step cascade model (TSCM), that limits the propagation to three-layer neighbors, and they experimentally verified that the model is suitable for simulating information propagation on Sina Weibo, a social site similar to Twitter. Then, they proposed an algorithm for solving the IM problem based on the TSCM. The above study draws on the three-degree influence theory [35], which we also consider in this work. The above studies show that a reasonable diffusion model that can accurately simulate information propagation on social networks is the key to effectively solving the IM problem. Most existing diffusion models have one commonality: the information spread between a pair of activated and inactivated nodes is independent of the states of their neighbors, and the accumulation of synergism has been neglected in these threshold models. Therefore, the scale of information diffusion is sensitive to the average degree of the network. Some studies show that parameter uncertainty may greatly affect influence maximization performance, and the interaction of combined nodes produces a collective influence that is larger than the sum of the individual nodes, which is called synergism [36–40]. Synergism is a ubiquitous phenomenon in social systems. Many studies have found that synergism enhances the transmission probability between a pair of nodes and promotes explosive spreading [29,41]. For example, in terms of information spread in social networks, a message transmitted by a group of connected users is more credible than a message transmitted by an individual [42,43]. Therefore, in this paper, we first discuss the catalytic role of synergism on the spreading dynamics in social networks and then propose a novel diffusion model called the synergism-based three-step cascade model (TSSCM) based on the above analysis and three-degree influence theory [35]. Finally, we develop an algorithm for solving the IM problem with the TSSCM. Experiments on five real networks demonstrate the efficacy of our method.

## Synergism-based three-step cascade model

### Definition of TSSCM

Without loss of generality, we define an unweighted, undirected graph *G* = (*V*,*E*), where *V* is the set of vertices and *E* is the set of edges. An online social network can be modeled by the graph. A node *v*∈*V* represents an individual in the social network, and an edge *e*(*u*,*v*)∈*E* denotes that information can spread between u and v. The topology is represented by the adjacency matrix {*A*_*ij*_}_*N*×*N*_, where *A*_*ij*_ = 1 if i and j are connected, and *A*_*ij*_ = 0 otherwise.

Many studies have found that synergism enhances the transmission probability and promotes explosive spreading [40]. Therefore, in our model, the probability that a seed node activates its neighbors is proportional to the number of other activated nodes connected to the seed node. Furthermore, some real information diffusion findings have supported the hypothesis that influence gradually dissipates and ceases to have a noticeable effect on people beyond the social frontier of three degrees of separation, which is called intrinsic decay [34,35,44,45]. Many research results on real social networks have confirmed this theory. Qin et al. [34] analyzed Sina Weibo retweet activities and illustrated that the retweet trees are small and shallow, and the average number of retweets decreases as the cascade depth increases. More than 96% of retweets are within three steps, and no retweet tree has deeper than 11 steps. Leskovec et al. [44] crawled blog links and found that more than 98.8% of the linked trees of all blogs have depths of less than three. Goel et al. [45] described the diffusion patterns arising from seven online social networks, including communications platforms, networked games and microblogging services, and found that most adoptions occur within a few steps of the seed node, even for the largest cascades observed. Based on the above research results, we consider the diffusion process within three steps and propose TSSCM.

In TSSCM, we suppose that node *u* can influence node *v* only if the distance from *u* to *v* is no greater than three. When *u* attempts to activate *v*, the activation probability *α*(*u*,*v*) is dependent not only on the number of activated neighbors connected to *u* but also the cascade depth *d*(*d* = 1,2,3).

α(u,v)=p(u,v)l(d) (1)

$$p(u,v)=1-{\left(1-\beta \right)}^{1+\frac{m}{k-1}}$$ (2)

$$l(d)=\frac{1}{d}(d=1,2,3)$$ (3)

where *β* is the basic spreading probability, *m* and *k* represent the number of activated neighbors connected to *u* and the degree of node *u*, respectively, *p*(*u*,*v*) represents the synergism spreading probability, and *l*(*d*) is the information decaying ratio, which is inversely proportional to *d*.

Eq (2) indicates that the larger the value of $\frac{m}{k-1}$, the higher the synergism spreading rate. Our model reduces to the classic decade model for *d* = 1 and *m* = 0, where *α*(*u*,*v*) = *β*. If *m*>0, then *α*(*u*,*v*)>*β*, which means synergism promotes information spread. In addition, *k*>1 for nonleaf nodes; thus, the synergistic ability of any activated neighbor of an active node is less than that of itself. This assumption is based on real disease propagation, where the probability that a susceptible node is infected by an infected direct neighbor is always greater than the probability of becoming infected from an infected indirect neighbor [36,37].

For *d*>1, we describe TSSCM as follows. Let *S*_0_⊆*V* be the seed set. All nodes in *S*_0_ are activated in the first time step. In the cascade steps 0≤*t*≤3, *S*_*t*_⊆*V* is the set of nodes that are activated at step *t*. At step *t*+1, each node *u*∈*S*_*t*_ attempts to activate its neighbor node *v*∉*S*_*t*_ with probability *α*(*u*,*v*). If such activation is successful, then *v* changes state from inactive to active and remains in the active state. Each activated node has only one chance to activate its neighbors during the step immediately following its initial activation. The above cascade process is repeated until no nodes in the network can be activated or *t* = 3.

**Fig 1:**
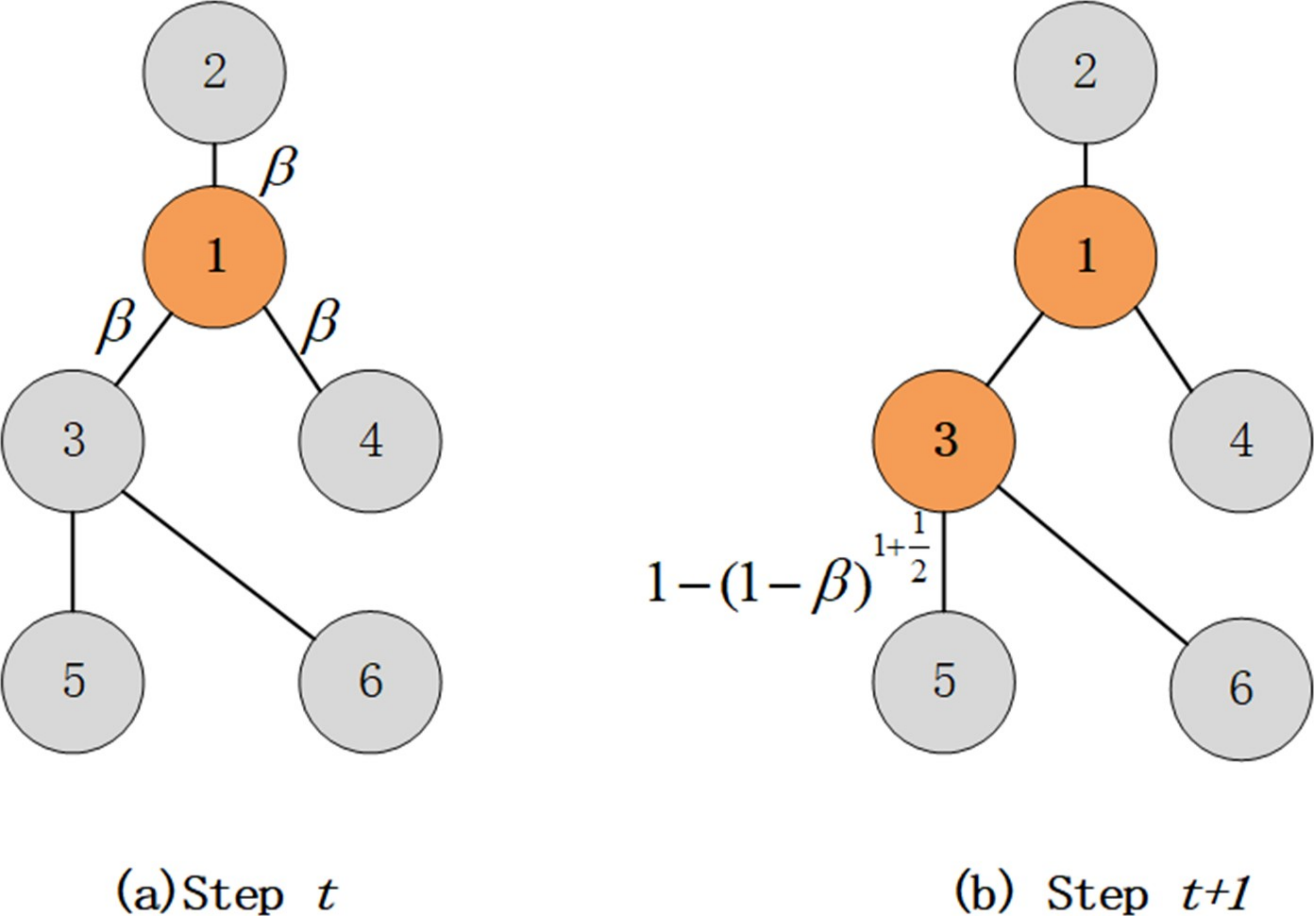
Illustration of TSSCM.

As shown in Fig 1, at step *t*, node 1 is a seed and the other nodes are inactive. For node 1, there are no active neighbors; thus, *m* = 0, $\alpha (1,2)=\alpha (1,3)=\alpha (1,4)=1-{\left(1-\beta \right)}^{1+\frac{0}{3-1}}=\beta$, which means node 1 activates its neighbors with probability *β*. At step *t*+1, node 3 is activated by node 1. Because it has an active neighbor, node 3 will activate its neighbors, node 5 and node 6, with a large probability $\alpha (3,5)=\alpha (3,6)=1-{\left(1-\beta \right)}^{1+\frac{1}{2}}$. Unlike other diffusion models, TSSCM accumulates synergism, i.e., active neighbors of an active node cooperate to spread information. This phenomenon is common in real social systems, such as microblogging retweeting [44], opinion propagation [30], and animal invasion [46].

### Influence maximization problem under TSSCM

Given a seed set S, we use *σ*_*TSSCM*_(*S*) to represent the influential spread of S, which can be quantified as the number of activated nodes under TSSCM when the propagation process ends. The IM problem under TSSCM is defined as follows.

**Definition 1** Given a network *G* = (*V*,*E*), the IM problem under TSSCM aims to find a subset S*⊆*V*, |S*| = *k* such that

$$S^{*}=\arg_{s}\;\max\sigma_{TSSCM}(S)$$ (4)

Kempe et al. [12] have proved that the IM problem is NP-hard using the maximum coverage problem. Inspired by this proof we consider a similar reduction method and prove that the IM problem under TSSCM is NP-hard.

**Theorem 1:** The IM problem under TSSCM is NP-hard.

**Proof:** The problem can be viewed as a Maximum Coverage problem, which is defined as follows:

Given a ground set *U* = {*u*_1_,*u*_2_,…,*u*_*n*_} and a collection of subsets *S* = {*S*_1_,*S*_2_,…,*S*_*m*_}, where *S*_*i*_⊆*U* and $\underset{i=1,2,..,m}{\cup }S_{i}=U$, we want to find *k* of the subsets *S*' = {*S*_1_,*S*_2_,…,*S*_*k*_}, *k*<*n*<*m*, where the union of *S*_*i*_⊆*S*',is equal to *U*. We show that the above description can be viewed as a special instance of the IM problem under TSSCM.

We define a directed bipartite graph containing *n*^2^+*m* nodes. Node *v*_*i*_ and node *v*_*j*_ correspond to *S*_*i*_ and *u*_*j*_, respectively. For each set *S*_*i*_, there is a corresponding node *v*_*i*_, and for each element *u*_*j*_, there are *n* corresponding nodes $v_{j_{1}},v_{j_{1}},\ldots ,v_{j_{n}}$. If *u*_*j*_∈*S*_*i*_, a direct edge $(v_{i},v_{j_{l}}),l=1,2,\cdots ,n$ exists with a spreading probability *p* = 1. We define *X* as a set of *k* of *S*_*i*_ and *T* as the union of the elements covered by *S*_*i*_∈*X*, *T*⊆*U*. If *k* nodes corresponding to *X* are selected, they activate the nodes corresponding to elements in *T*; then, the number of active nodes is *k*+*n*|*T*|. Similarly, in the converse direction, the number of active nodes is also *k*+*n*|*T*|. In summary, we know that the maximum coverage instance |*T*| element can be covered by *k* sets if and only if *k*+*n*|*T*| nodes can be activated by *k* seeds in the instance, i.e., *σ*_*TSSCM*_(*X*) = *k*+*n*|*T*|. In the instance, the longest path of the directed bipartite graph includes two nodes; thus, in TSSCM, *l*(1) = 1 and *l*(2) = *l*(3) = 0. *k* active seed nodes correspond to a maximum coverage solution due to information propagation to all other nodes corresponding to the ground set *U*. Thus, the maximum coverage problem is solved.

The optimal solution of the IM problem under TSSCM can be approximated by the greedy algorithm, *Greedy*(*G*,*σ*_*TSSCM*_(*S*),*k*), as shown in Algorithm 1.

**Algorithm 1** *Greedy*(*G*,*σ*_*TSSCM*_(*S*),*k*)

Input: *G*: network; *k*: size of seed set

**Output: seed set** *S*

**1: initialize** *S* = ∅

**2: while** |*S*|<*k* do

**3:    select** *v* = arg max_*v*∈*V*_(*σ*(*S*∪*v*)−*σ*(*S*));

**4:**    *S* = *S*∪*v*;

**5**: **end while**

**6: return** *S*;

The approximation ratio of the greedy algorithm can reach $1-\frac{1}{e}\approx 0.63$

To optimize the global function of the IM problem, Flaviano Morone [30] mapped information spread asymptotically onto the optimal percolation and proposed another topological centrality measure called CI, which is defined as

$$CI_{l}(i)=(k_{i}-1)\underset{j\in \partial Ball(i,l)}{\sum }(k_{j}-1)$$ (5)

where *k*_*i*_ is the degree of node *i*, and ∂*ball*(*i*,*l*) denotes the set of nodes at a distance *l* from node *i*.

**Fig 2:**
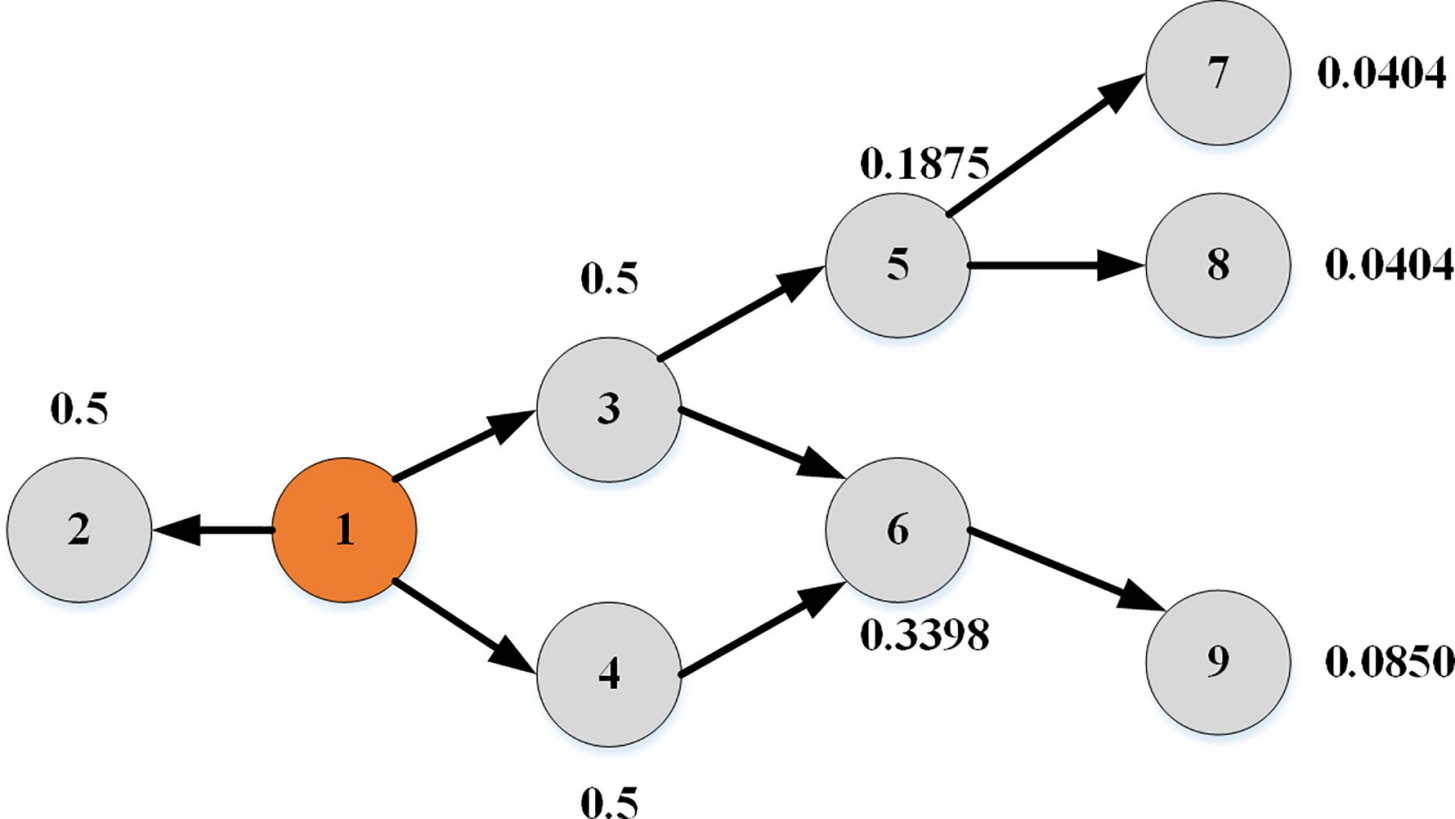
The final activation probabilities of the nodes.

The above CI does not consider the spreading rate between two linked nodes. However, in the actual information spreading process, a node receives information transmitted by other neighbor nodes with a certain probability. Clearly, a realistic and efficient algorithm for optimal resource allocation should consider both the topological characteristics and the details of the dynamics; additionally, propagation should be maximized within a limited time window. Because TSSCM is inherently probabilistic, we proposed a measure, called three-layer collective influence with synergism (CI_TLS), that incorporates CI formulation and spreading dynamics with synergism. For node *i*, *l*(*i*,*v*) denotes the shortest distance from *i* to *v*. The spreading influence of node *i* is confined to a node set that consists of the nodes at a distance *l* from *i*, *L*(*i*,*l*) = {*v*|*l*(*i*,*v*) = *l*,*v*∈*V*. We assign to node *i* the CI_TLS following Eq (6):

$$CI\_TLS(i)=\underset{v\in L(i,l)}{\sum }\sigma (i,v)\;l=1,2,3$$ (6)

$$\sigma (i,v)=1-\prod_{\begin{array}{l} u\in L(i,l-1)\\ A_{uv}=1 \end{array}}(1-\sigma (i,u)\alpha (u,v))$$ (7)

where *α*(*u*,*v*) is the activation probability defined in Eq (1). *σ*(*i*,*v*) is the final activation probability of node v by node *i*, which is obtained by recursively calculating the influence propagation. The CI_TLS of a node contains rich topological information and propagation dynamics, which can tell us more about the roles of the nodes in the network than a measure that considers only one aspect. In Eq (6), the sum contains the contributions of nodes whose distance to *i* is less than 3. Therefore, a node located at the center of a cluster with many links would have a large CI_TLS, even if it has a low degree. Thus, these low-degree nodes with the bridging property outrank those with a large degree but mediocre peripheral location. Fig 2 provides an illustrative example. We set node 1 as the initial seed, *β* = 0.5. The number next to node *v* is the value of *σ*(1,*v*). The arrows denote the direction of information spread. The calculation is as follows.

$$\begin{array}{l} \sigma (1,6)=1-[1-\sigma (1,3)\alpha (3,6)][1-\sigma (1,4)\alpha (4,6)]\\ =1-\left[1-0.5*\left(1-{\left(1-\beta \right)}^{1+\frac{1}{2-1}}\right)*\frac{1}{2}\right][\left[1-0.5*\left(1-{\left(1-\beta \right)}^{1+\frac{1}{2-1}}\right)*\frac{1}{2}\right]\\ =1-\frac{13}{16}*\frac{13}{16}\\ =0.3398 \end{array}$$

Node 6 has a larger activation probability than node 5 because node 6 is influenced by two nodes while node 5 is influenced by one node. Similarly, the activation probability of node 9 is larger than those of nodes 7 and 8 because node 6, which precedes node 9, has two activated neighbors, while the preceding node of nodes 7 and 8 has one activated neighbor. Therefore, the synergistic influence on node 9 is greater than that on nodes 7 and 8. *CI*_*TLS*(1) is the sum of *σ*(1,*i*),*i* = 2,3,4,5,6,7,8,9, and it is used to measure the spreading influence of node 1 in CL_TSL.

We propose an adaptive CI_TLS algorithm based on the greedy approach to obtain a scalable algorithm. Define *N*(*i*,4) as the set of node *i* plus those nodes with a short distance to *i* of less than 4. The details are shown in Algorithm 2.

**Algorithm 2** *CI*_*TLS*(*G*,*k*)

**Input:** *G***: network;** *k***: size of seed set**

**Output: seed set** *S*

**1: initialize** *S* = ∅

**2: Calculate** *CI*_*TLS*(*i*) for each node i

**2: while** |*S*|<*k* **do**

**3:    select** *i* = arg max_*i*∈*V*_*CI*_*TLM*(*i*);

**4:**    *S*= *S*∪*i*;

**5:** Remove *N*(*i*,4) and decrease the degree of *N*(*i*,4)’s neighbors by 1.

6: Update *CI*_*TLS*(*i*) for all nodes

**7**: **end while**

**8: return** *S*;

Note that we remove *N*(*i*,4) once *i* is added to the seed set *S* (line 5 in algorithm 2) because seed *i* activates *N*(*i*,4); thus, the nodes in *N*(*i*,4) do not have to be selected in the later calculation. Ideally, *N*(*i*,4) can be identified during the computation of *CI*_*TSL*(*i*) without additional time. The above operation overcomes the defect of the traditional algorithm, where the influence areas of the selected seeds overlap.

### Computational complexity analysis of our algorithm

Next, we demonstrate the efficiency of our algorithm by investigating its computational complexity. In a network with *N* nodes, to compute the CI_TLS of a node, we must iteratively traverse its neighbors within a finite search radius, which costs *O*(〈*k*〉), where 〈*k*〉 is the average degree of the network. Because *k*≪*N*, the result is *O*(1). CI_TLS must be calculated for every node in the first step. However, during later steps, we have to recalculate the values of only the nodes within *l*+1 layers of the removed nodes. As verified in reference [31], the computational complexity of the above problem is *O*(1), compared to *N*→∞. Sorting *CI*_*TSL*(*i*) requires *O*(*N*log*N*), and we select nodes until the seed set includes k nodes; therefore, the total computational complexity of our algorithm is *O*(*kN*log*N*), which ensures that our algorithm is scalable to large networks.

## Experiments

First, we analyze the retweet data from Sina Weibo and verify that the proposed synergism-based TSSCM can effectively simulate real propagation trends. Then, we compare the spreading influence of the seed sets obtained by the five IM algorithms under TSSCM and the independent cascade model (ICM) to test the effectiveness of the CI_TLS algorithm. Finally, we list the CPU times of the five algorithms.

### Analysis of the real diffusion depth

We obtained the post and retweet data from May 3–11, 2014, based on the Sina Weibo API (http://open.weibo.com). In accordance with the breath-first strategy, we crawled 50 messages posted by a user, and for each message, crawled the retweet users and added them to the crawling queue. After processing a user, we removed the user from the queue to reperform the same operational process and loop back and forth. Finally, we randomly selected 2000 retweet trees.

**Fig 3:**
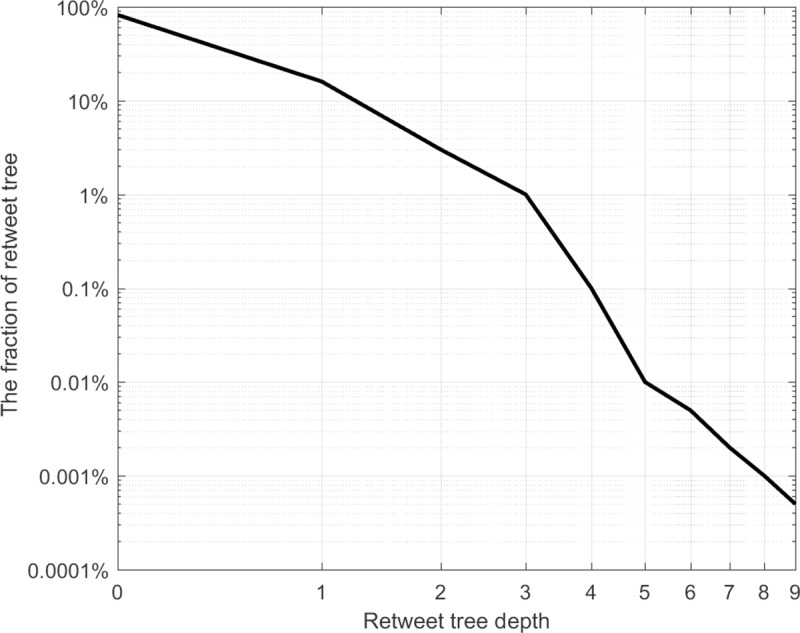
The fractions of retweet trees.

We counted the fraction of retweet trees at each depth. The results are shown in Fig 3. We can observe that the retweet trees are all small and shallow, and the number of retweet trees decreases as the cascade depth increases. Fig 3 shows that most of the cascades are within three steps and that less than 1% of retweet trees have a depth beyond 3. The fraction of retweet trees deeper than 8 is only 0.01% of all trees. Other researchers obtained similar conclusions, such as in references [34,35,44,45].

### Datasets used in the experiments

Table 1 lists the empirical networks used to evaluate the effectiveness and efficiency of our proposed algorithm, namely, Blog, DBLP, Email, Epinions, Twitter and Livejournal, all of which can be downloaded from http://networkrepository.com[47].

**Table 1:** The statistical properties of the six empirical networks^a^.

Network	*n*	*m*	*k*_max_	〈*k*〉	*C*	*β*_*th*_
Blog	10K	326K	3992	64.78	0.0914	0.0018
DBLP	317K	1M	343	6.62	0.6350	0.0834
Email	1K	5K	71	9.62	0.2202	0.0535
Epinions	27K	100K	443	7	0.1351	0.0758
LiveJournal	4M	28M	3k	13	0.2600	0.0534
Twitter	405K	713K	626	3	0.014	0.1874

a. http://networkrepository.com

In Table 1, *n* and *m* are the total numbers of nodes and edges, respectively; *k*_max_ and 〈*k*〉 represent the maximum and average degrees, respectively; *C* is the clustering coefficient; and *β*_*th*_ is the epidemic threshold. In homogenous networks, $\beta_{th}=\frac{1}{\langle k\rangle }$, while in heterogeneous networks, $\beta_{th}=\frac{\langle k\rangle }{\langle k^{2}\rangle }$ [26].

### Baseline algorithm

We choose four algorithms, namely, CI, degree discount, MaxCoreCover and random, as the baselines for evaluating the performance of our algorithm.

CI: CI, which is defined as Eq (5), was proposed by Flaviano and Hernan A [30]. *CI*_*l*_ is adaptive and achieves the best performance for *l* = 3,4. In this paper, we choose *CI*_4_ as a baseline algorithm.

Degree Discount: The degree discount algorithm is a heuristic based on degree centrality [23]. The node with the largest degree is selected as a seed, and the degrees of its neighbors are discounted by 1.

MaxCoreCover: This algorithm, which selects the node with the largest k-shell as a seed, was proposed by Kitsak [21]. When a node is selected, its neighbors can no longer be seeds.

Random: This algorithm randomly selects seed nodes.

### Evaluation methodologies

The evaluation indicators we adopt for the IM algorithms are as follows: (a) the spreading influence of the seed set for TSSCM, (b) the spreading influence of the seed set for ICM, and (c) the computational time required by the IM algorithm to find the seed set.

**Fig 4:**
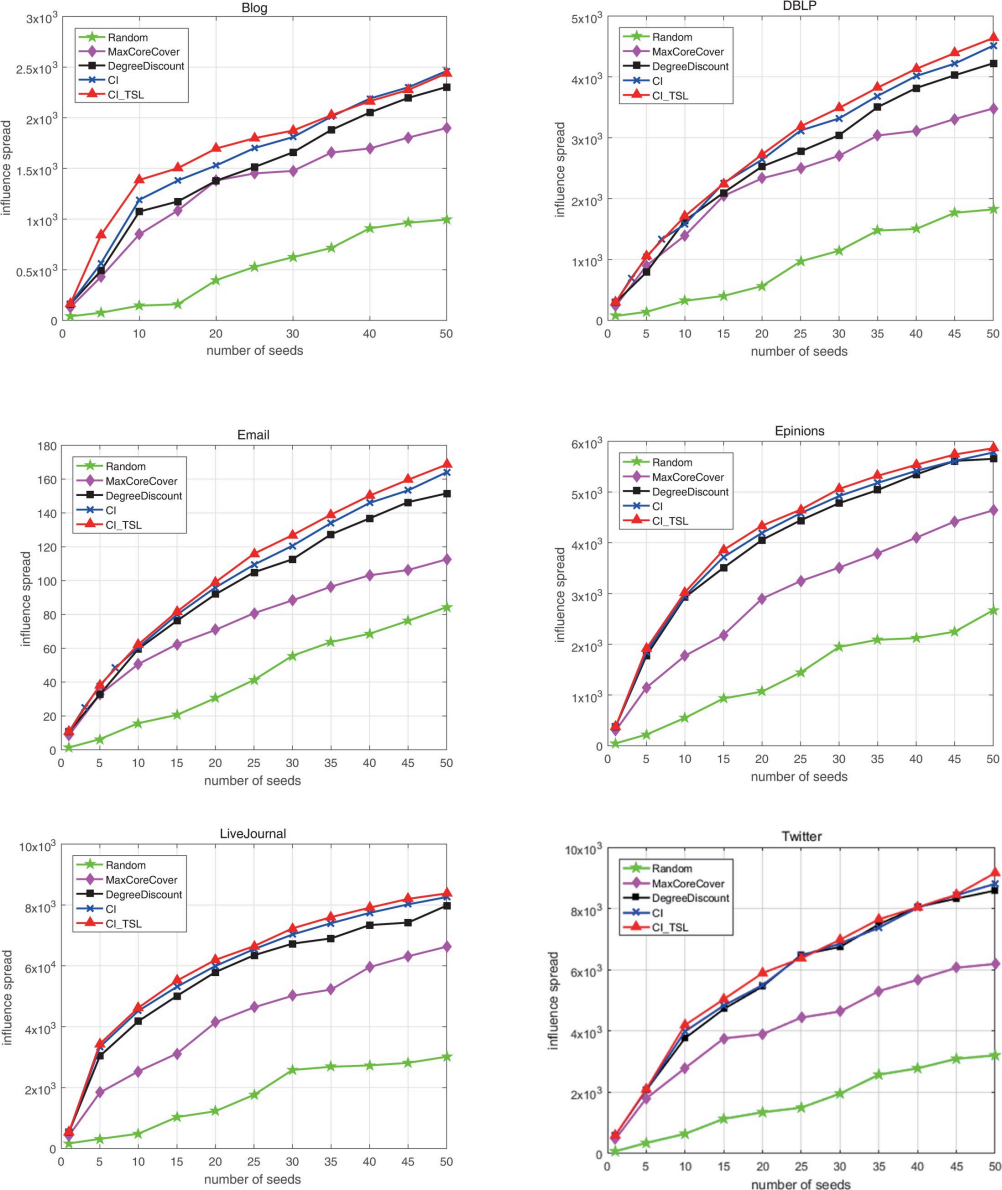
Spreading influence results of five algorithms for six networks.

The spreading influence of a seed set, which is used to evaluate the performance of an IM algorithm, is defined as the number of active nodes after the propagation process is complete. The larger the spreading influence is, the more accurate the algorithm. In this paper, we first selected the seed set of each network according to the five algorithms. Then, we compared the spreading influence of different seed sets using five algorithms for each network under TSSCM and ICM. We have shown that the IM problem under TSSCM is NP hard; therefore, we run 10000 Monte Carlo simulations to obtain the results. The five measures are compared in Fig 4, which shows the spreading influence of the seed sets selected by these measures in six real networks. The x-axis represents the number of seeds obtained in the first step, and the y-axis represents the spreading influence of the five algorithms for a network, i.e., the number of active nodes after propagation is complete. The basic spreading probability *β* = *β*_*th*_, and the values of *β*_*th*_ are listed in Table 1.

As expected, the seed sets obtained by CI_TLS result in the widest information spread, which means the performance of CI_TLS is the best. The trend lines of degree discount and CI are similar to those of CI_TLS because these three algorithms account for the number of neighbors when selecting seed nodes. The performance of CI ranks second among the five measures because CI_TLS considers the effect of synergies between nodes on the propagation probability while CI does not, which validates the rationality and importance of synergy. Degree discount does not consider dynamic attributes, such as the propagation path and the spreading probability between nodes, but considers static attributes, such as node degree, which results in performance that is inferior to those of CI_TLS and CI. However, degree discount performs much better than MaxCoreCover, which indicates that the degree of a node is an important indicator of the node’s influence. In addition, the pruning strategy adopted in this measure ensures that the selected seed nodes do not gather in a local area of the network; this strategy is also used in CI_TLS. The random algorithm always has the worst results, indicating that careful seed selection is indeed important for effectively identifying influential nodes in many applications, such as marketing campaigns, epidemic prevention and maximization of information spread.

The spreading probability *β* is a parameter of TSSCM, and different values of *β* will result in different diffusion processes. Next, we compare the performance of different algorithms for the DBLP based on TSSCM with different *β*, where *β*∈{0.04,0.05,0.06,0.07}.

**Fig 5:**
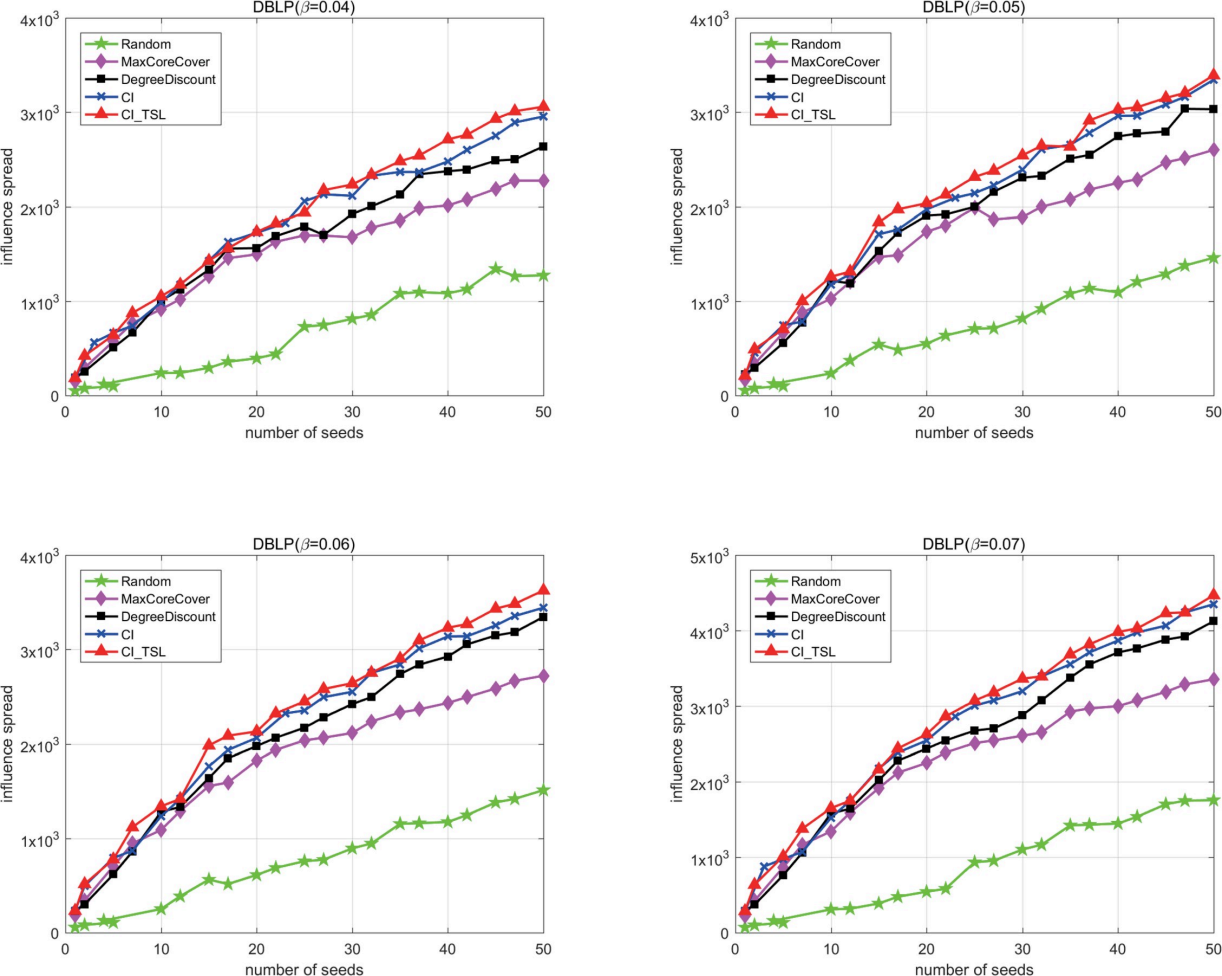
The spreading influence of different algorithms on the DBLP based on TSSCM with different *β* values.

Fig 5 shows that the performance of CI_TLS is better than that of the other four algorithms over the entire range of *β*. At the same spreading probability, when the number of seeds is less than 5, the diffusion ranges of these algorithms are similar, except that of Random. Notably, the spreading influence of a few seeds is very limited. As the number of seeds increases, the performance gap of the five algorithms becomes obvious. Next, we analyse the results with different spreading probabilities. As *β* increases, the superiority of the three degree-based algorithms, CI_TLS, CI, and DegreeDiscount, becomes increasingly obvious, and CI_TLS always performs best. These results indicate that the degree is a key attribute of a node. CI_TLS considers and improves the degree measure by adding spreading distance constraints and a collaborative promotion mechanism; thus, it can mine influential nodes more effectively than can other other methods.

**Fig 6:**
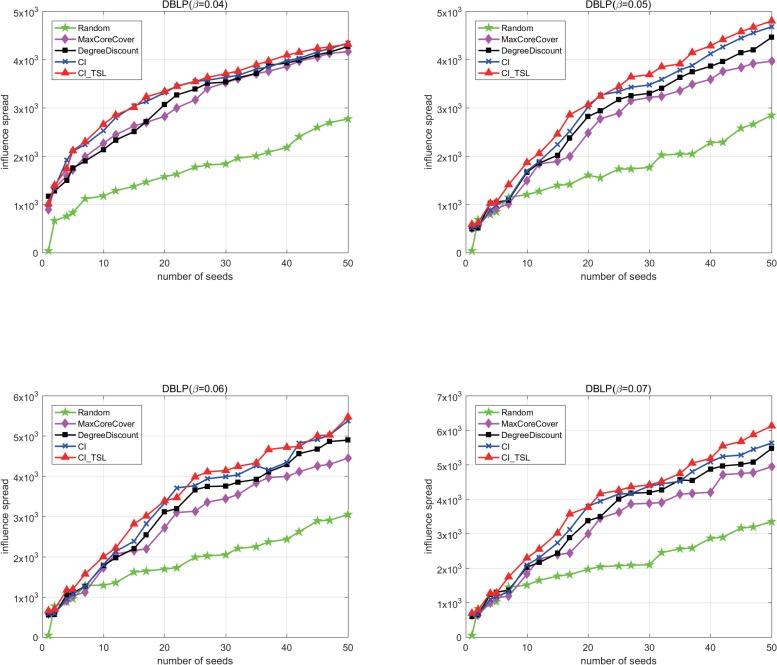
The spreading influence of different algorithms on the DBLP based on ICM with different *β* values.

To further verify the performance of the proposed algorithm, we compare the spreading influence of seed sets based on ICM which is widely used in Influence maximization problem, and show the simulate results of the five algorithms in Fig 6. The CI_TLS shows its advantages over the whole range of *β*, *β*∈{0.04,0.05,0.06,0.07}. Formula (6) shows that a node with many activated neighbors can easily spread information to its inactive neighbors, that is, a node with many k-step connected neighbors would have a wide propagation range. The synergy mechanism in the CI_TLS makes it possible to effectively find such nodes, which is why the algorithm displays excellent performance.

**Fig 7:**
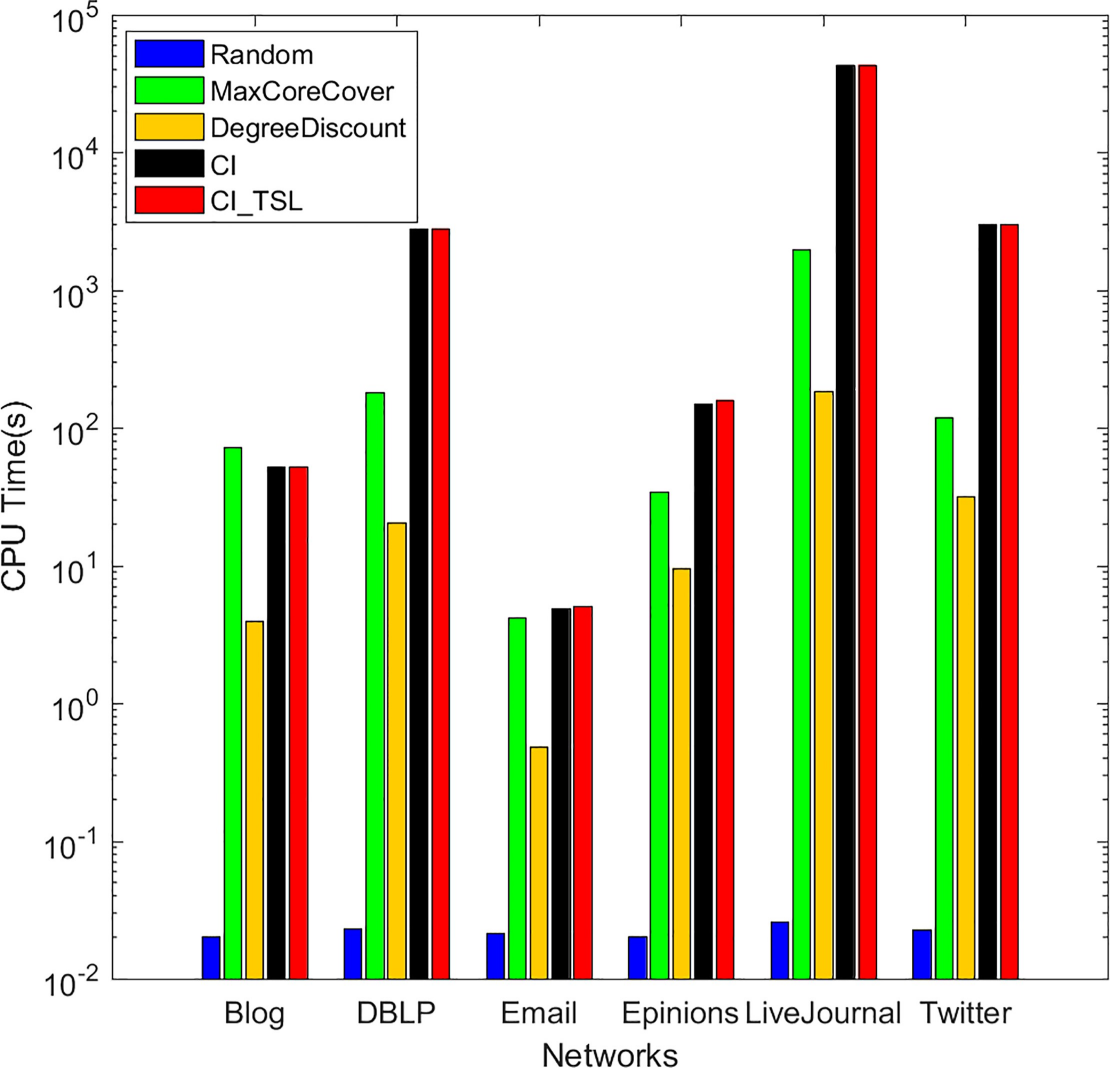
CPU times of five algorithms on six networks.

Finally, we compare the computational times of the five algorithms. The experiments are run on a server with a 4-core processor and 32 GB RAM using Python. Table 2 shows the computation times required by the five algorithms to find 50 seeds on six networks, i.e., Blog, DBLP, Email, Epinions, LiveJournal and Twitter. The computation time of our algorithm is longer than those of random, MaxCoreCover and DegreeDiscount, but it is almost equal to that of CI on all six real networks. Compared with CI, our method has a computation time increase of less than 2%. The CPU times of these algorithms are compared in Fig 7, where the x axis represents the algorithms and the y axis represents the computation times required to obtain 50 seeds. We can see that our algorithm is suitable for large-scale networks and can effectively mine influential nodes.

**Table 2:** The CPU times (in seconds) of five measures for six networks.

Network	Random	MaxCoreCover	DegreeDiscount	CI	CI_TLS
Blog	0.0201	72.5811	3.9826	52.6322	52.7215
DBLP	0.0231	180.9127	20.7354	2803.154	2820.4001
Email	0.0214	4.1752	0.4882	4.8861	4.9652
Epinions	0.0204	34.5144	9.6482	150.4257	152.8561
LiveJournal	0.0258	1975.6480	186.3461	43406.4718	43510.5842
Twitter	0.0226	120.1831	32.0974	3000.1289	3013.0084

Overall, from the results shown in Figs 4, 5, 6 and 7, the proposed algorithm is more efficient in solving IM problems than the other four algorithms.

## Conclusion

Solving the IM problem is important for network analysis, information spreading, and other applications. A new diffusion model based on three degrees of influence theory and the catalytic role of synergism on spreading dynamics, namely, TSSCM, is proposed in this paper. In our model, the probability that a seed node activates its neighbors is proportional to the number of activated nodes connected to the seed node, which is called synergism. Moreover, our model accurately simulates the cascade process of information transmission with finite steps. Inspired by the CI algorithm, we devised an algorithm for solving the IM problem under TSSCM, namely, CI_TLS. Compared with the CI algorithm, CI_TLS adds only the calculation of the activated neighbors of a node; therefore, the computation time increases only slightly, thereby balancing computational complexity and precision. The experimental results on six networks show that the CI_TLS measure is better than the other four algorithms tested, i.e., random, MaxCoreCover, degree discount and CI, and it achieves the best results for mining influential nodes. The seed sets obtained by CI_TLS result in the widest information spread. With the scale of social networks growing continuously, we can use parallel computing to accelerate the algorithm to effectively and efficiently solve the IM problem in large-scale networks. In many social networks, user behavior is affected by psychological factors, and a diffusion model with user decision making based on game theory would be appropriate [48–50]. Further work could track the IM problem under a diffusion model with psychological game theory. In reality, each individual in a network is always a user in the other networks. Resource diffusion impacts epidemics and information spread [51], and thus, a synergism-based diffusion model in multiple networks would be interesting and important to evaluate in future research.
